# *NUAK2* Pathogenic Variants Are Definitively Associated with Neural Tube Defects in Humans: New Genotype-Phenotype Correlation and Review of the Literature

**DOI:** 10.3390/diagnostics15182289

**Published:** 2025-09-10

**Authors:** Gioia Mastromoro, Claudio Dello Russo, Stefania Mariani, Serena Bucossi, Riccardo Riccardi, Amit Pal, Rosanna Squitti, Mehak Dangi, Antonio Pizzuti, Mauro Ciro Antonio Rongioletti

**Affiliations:** 1 Department of Laboratory Science, Research and Development Division, Ospedale Isola Tiberina—Gemelli Isola, 00186 Rome, Italy; claudio.dellorusso.fw@fbf-isola.it (C.D.R.); stefania.mariani.fw@fbf-isola.it (S.M.); serena.bucossi.fw@fbf-isola.it (S.B.); maurociroantonio.rongioletti@fbf-isola.it (M.C.A.R.); 2 Unit of Molecular Genetics, Center for Advanced Studies and Technology, ‘G. d’Annunzio’ University of Chieti-Pescara, 66100 Chieti, Italy; 3 Neonatology Unit, Ospedale Isola Tiberina—Gemelli Isola, 00186 Rome, Italy; riccardo.riccardi@fbf-isola.it; 4 Department of Biochemistry, AIIMS, Kalyani 741245, India; maximus1134@gmail.com; 5 Department of Theoretical and Applied Sciences, eCampus University, 22100 Novedrate, Italy; 6 Centre for Bioinformatics, Maharshi Dayanand University, Rohtak 124001, India; mehak.bioinfo@mdurohtak.ac.in; 7 Department of Experimental Medicine, Sapienza University of Rome, 00185 Rome, Italy; antonio.pizzuti@uniroma1.it

**Keywords:** neural tube defects, sacral dimple, *NUAK2*, spinal dysraphism, prenatal diagnosis

## Abstract

**Background and Clinical Significance:** Neural tube defects (NTDs) represent a group of malformations, typically arising from a complex interplay between genetic susceptibility and environmental influences. Increasing evidence points to the contribution of rare pathogenic variants in genes involved in embryonic development in selected cases. To date, two families with NTDs carrying biallelic variants in *TRIM36* and *NUAK2* have been described. Specifically, germline homozygous pathogenic variants in *NUAK2* were identified in three fetuses with anencephaly, thus implicating this gene as a critical regulator of neural tube closure. **Case Presentation:** We describe a family in which five individuals presented with sacral dimples, a subtle midline defect considered a minor malformation. Exome sequencing revealed a heterozygous missense variant, c.487G>A in *NUAK2*, segregating with the phenotype. Although sacral dimples are often clinically silent and do not typically cause functional impairment, their presence in multiple relatives highlights a possible shared genetic etiology. Careful phenotypic recognition of such findings can therefore provide valuable insights into underlying molecular mechanisms. **Conclusions:** This report extends the clinical spectrum of *NUAK2*-related anomalies by demonstrating a novel genotype–phenotype correlation. Our findings suggest that variants in this gene may follow a semi-dominant inheritance pattern, with heterozygous carriers manifesting milder phenotypes, such as sacral dimples, while biallelic pathogenic variants lead to severe NTDs. This observation reinforces the association between *NUAK2* loss-of-function variants and NTDs and emphasizes the importance of genetic investigations in families where such dysmorphic traits recur. Ultimately, these results contribute to clarifying the molecular basis of NTDs and may inform both genetic counseling and risk stratification in affected families.

## 1. Introduction

Neural tube defects (NTDs) occur in 0.5–2 out of 1000 pregnancies worldwide. These developmental anomalies are generally classified according to the site of the five where the neural tube closure failed to occur, three in the cranial and two in the caudal region [1]. They bear a moderate risk of miscarriage.

Prenatal ultrasound exhibits high specificity and high accuracy across all trimesters, with higher sensitivity in the second trimester.

First-trimester ultrasound performed between 11w3d and 13w6d allows for the evaluation of the thickness of nuchal translucency and to detect approximately 37.5% of major malformation overall [2,3,4,5]. During the second trimester, systematic scans between 18 and 24 gestational weeks yield substantially higher detection rates for many structural anomalies. Sensitivity varies by organ system, being highest for thoracic and abdominal wall anomalies and lowest for gastrointestinal defects [5]. In the third trimester, the incremental detection of new major anomalies is lower, as many anomalies are already identified earlier. Postnatal detection rates of malformations exceed the prenatal yield (7.24% versus 2.95% prevalence), highlighting limitations of ultrasound across trimesters [6].

Anencephaly can be diagnosed by ultrasound in the late first trimester [2]. It is the most frequent NTD [7] and presents a recurrence risk of 2–5% following a single case, even if in isolated populations it has been described as a single-gene disorder with an autosomal recessive trait of inheritance [8,9]. After the failure of the closure of the rostral neuropore, cells usually contained in the neural tube are exposed to amniotic fluid and degenerate [10], determining the absence of the cranial vault and intracranial tissues.

Spinal dysraphisms occur as a result of caudal closure defects and include a range of conditions with a spectrum of signs and symptoms of varying severity.

NTDs, especially myelomeningocele, are managed surgically to reduce infection and neurological deterioration [11]. While postnatal closure within 48 h is standard, the Management of Myelomeningocele Study (MOMS) trial demonstrated that prenatal repair before 26 weeks decreases shunt dependency and improves motor outcomes, though with higher maternal–obstetric risks [12]. Emerging fetoscopic techniques aim to balance fetal benefit with reduced maternal morbidity, but long-term data are pending [13].

Although NTDs are considered complex diseases, determined by the interaction of multiple environmental and genetic factors [14], it has been estimated that empiric recurrence risk after pregnancy with NTDs is 3% (an increase of more than 40-fold), and 10% after two occurrences [15,16].

NTDs have been associated with folate metabolism and Wnt signaling genes. The association between reduced folate levels and increased NTD risk has been evidenced since 1965 [17]. For this reason, as primary prevention, it is currently recommended that all women of reproductive age have an intake of 400 micrograms of folic acid daily. For women with a previous pregnancy with NTD, the intake is raised to 4000 micrograms starting from the time of planned conception, reducing their risk for NTD by 70% [18].

Recent experimental and clinical evidence suggests a potential role for inositol, a naturally occurring polyol involved in cell signaling and membrane biogenesis, in preventing folate-resistant cases.

Studies on animal models carrying alleles that result in loss or reduced expression of inositol kinases demonstrated that inositol supplementation can decrease the frequency of NTDs in folate-deficient mice, likely through modulation of phosphatidylinositol signaling pathways that regulate cell proliferation and morphogenesis [19]. In humans, pilot trials indicated that inositol, when administered in combination with folic acid, may lower the recurrence risk of NTDs in women with a previous affected pregnancy [20].

Origin in certain geographical areas (Northern Ireland, Northern China) [21,22,23], ethnicity, and comorbidities such as diabetes are considered predisposing factors. It is, therefore, impossible to define generic risk figures for the world’s general population. NTDs can also be induced by environmental factors, including hyperthermia, vitamin B9 or B12 deficiency, medical drugs like anticonvulsants and insulin.

Experimental evidence from genetic preclinical models of mutant mice highlighted the involvement of the planar cell polarity pathway. However, these findings still need to be confirmed by human phenotypes [10].

Some families in which NTDs recurred more than expected for a complex disease had been described [8,9], raising the suspicion of the existence of a Mendelian trait of inheritance. However, in most cases, a monogenic cause has not been identified.

We report on a Caucasian family including five individuals with a sacral dimple, segregating with a monoallelic variant in a gene previously described in fetuses with anencephaly [9].

We performed a literature review of NTD cases in which causative variants in genes associated with these phenotypes have been identified. We searched the PubMed database (https://pubmed.ncbi.nlm.nih.gov/), accessed on 25 August 2025, for (“neural tube defect” or “anencephaly” or “spina bifida” or “myelomeningocele”) AND (“cytogenetics” or “molecular genetics” or “genetics”), including prenatal and postnatal cases in which genetic tests yielded a diagnosis. Polymorphisms, cases carrying variants but without reported phenotype, and cases with biallelic variants reported in both affected and unaffected individuals of the same family were excluded.

## 2. Case Description

### 2.1. Family Assessment

**Figure 1:**
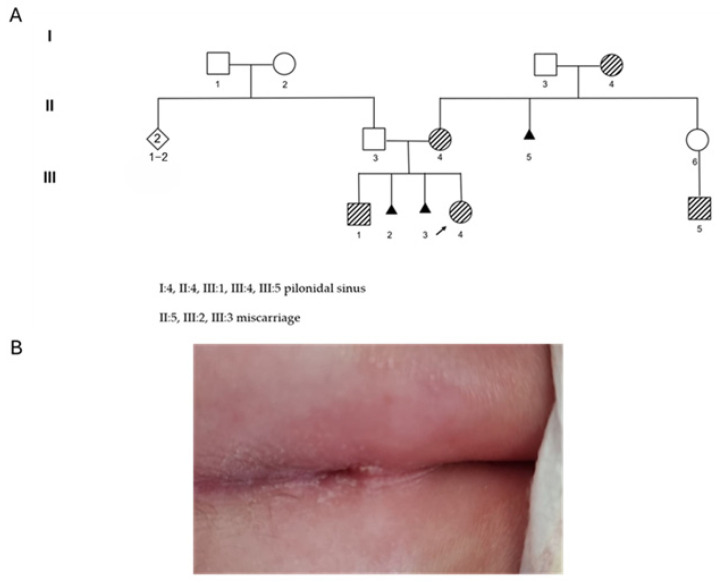
(**A**) Pedigree of the family presenting with a sacral dimple. The arrow (↗) highlights the proband; the black triangle (▲) shows miscarriage of the first trimester; the striped symbol (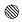) highlights the individual with the sacral dimple. Individuals I:4, II:4, III:1, III:4, III:5 show the phenotype. (**B**) Sacral dimple of the proband.

A female newborn (Figure 1A, III:4) was referred to genetic counseling because of the presence of hypertelorism and a deep sacral dimple above the gluteal cleft, without hypertrichosis and discoloration of the region (Figure 1B). Prenatal history was uneventful, with normal ultrasound scans. The neonate was born after vaginal delivery. During the counseling, the mother reported two miscarriages of the early first trimester without a known cause, and revealed that five individuals in the family presented with pilonidal sinus (Figure 1A, III:1, III:5, II:4, I:4). None of them showed related neurological consequences. Chromosomal Microarray Analysis (CMA) and exome sequencing were requested. The CMA performed on the neonate’s peripheral blood did not identify imbalances. Exome sequencing was performed on the genomic DNA of both the neonate (III:4) and maternal grandmother (I:4).

### 2.2. Exome Sequencing

Sequencing libraries for exome sequencing data analysis were prepared using the Cell3™ Target Whole Exome Enrichment Nonacus and NovaseqX (Illumina, San Diego, CA, USA). A minimum depth of coverage of 20X was obtained in coding sequences and splicing regions. Reads were mapped to the human genome (GRCh37) using BWA. BAM files were created using Picard tools and subjected to local realignment (using InDels and SNPs from 1000 Genomes) and base quality score recalibration using GATK. VCFs were filtered using FilterMutectCalls (GATK) and annotated with Variant Effect Predictor. First selection of the detected variants was performed for gene/phenotype association using Human Phenotype Ontology (HPO) and setting filtering for “Neural Tube Defect”. The population frequency was determined by the ExAC and GnomAD databases. Annotated variants were classified according to the criteria of the American College of Medical Genetics and Genomics [24]. For missense single nucleotide variants not described as pathogenic, Functional Analysis Through Hidden Markov Models (FATHMM), Combined Annotation Dependent Depletion tool (CADD v1.7) [25], MutationTaster were used as variant effect predictors. SIFT-indel was used to predict the functional impact of in-frame insertions and deletions. RegulomeDB (www.regulomedb.org, accessed on 4 December 2024 ) was used to assign the likely functional impact of non-coding variants, occurring in introns, upstream or downstream of coding regions in 3′ and 5′ UTRs, and in intergenic regions. To prioritize the variants, we used ClinVar, Mendelian Inheritance in Man (http://omim.org, accessed on 10 December 2024), and Human Phenotype Ontology (HPO) to classify variants having clinical significance as disease-causing. Gene Ontology (GO, https://amigo.geneontology.org/amigo/search/bioentity, accessed on 9 December 2024), was used to identify genes having a function that coincides with the pathology of the disease of interest. KEGG pathways (www.genome.jp/kegg/, accessed on 11 December 2024) were used to define gene products as associated with pathways involved in disease. Gene Expression Omnibus profiles (www.ncbi.nlm.nih.gov/geoprofiles, accessed on 14 January 2025) and the Expression Atlas (https://www.ebi.ac.uk/gxa/home, accessed on 13 January 2025) were used to discriminate genes expressed in the tissue or organ of interest.

### 2.3. Docking Analysis

**Figure 2:**
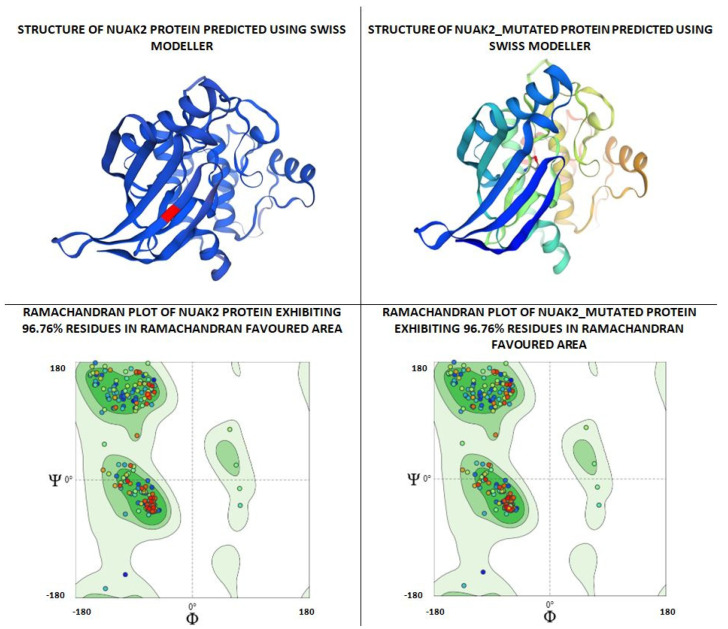
Depicting the pose of the predicted 3-D structure of normal and mutated NUAK2 protein. Ramachandran plot of the predicted structures of both proteins.

As per the PROSITE analysis, the kinase portion (53–303 residues) of the NUAK2 protein was identified, and its 3D structures for both normal and mutant proteins were predicted using the SWISS-MODELLER Server. The structures were further evaluated using Ramachandran plot and ERRAT scores, which suggested that the predicted structures are of high quality and can be used for docking analysis (Figure 2).

The structural comparison of the two predicted structures was performed using Pymol software version 3.1.4 for Windows, and the image of the structure alignment suggested that there is hardly any difference observed in the 2D and 3D structures of the two proteins. The Root Mean Square Deviation obtained on the structural alignment of both normal and mutated proteins was just 0.012 Å.

### 2.4. Results of Genetic Testing and Docking Analysis

CMA did not reveal genomic imbalances. Exome sequencing was performed on the peripheral blood of the female child (III:4) and grandmother (I:4), both presenting with a sacral dimple. After filtering for gene/associated HPO list, we obtained that variants in two genes were shared by both samples. In particular, the missense heterozygous c.487G>A; p.(Val163Met), NM_030952.3, rs752932851 variant in the *NUAK2* gene (MIM*608131; NUAK FAMILY, SNF1-LIKE KINASE, 2), and the c.*131_*136delGAGAGA variant, in the 3′UTR in the *RREB1* gene. These variants can be classified as “variants of uncertain significance” according to the American College of Medical Genetics and Genomics criteria. Segregation analysis detected the same *NUAK2* variant in the proband’s brother (III:1). In order to validate the effect of the detected variant on the NUAK protein, a docking analysis was performed.

The pattern of binding energies at the predicted ATP binding site (6–14 residues of the kinase domain) in normal and mutated proteins suggested that ATP binds with lower energy (−10.595 kcal/mol) in normal protein as compared to mutated protein (−8.881 kcal/mol) (Table 1).

**Figure 3:**
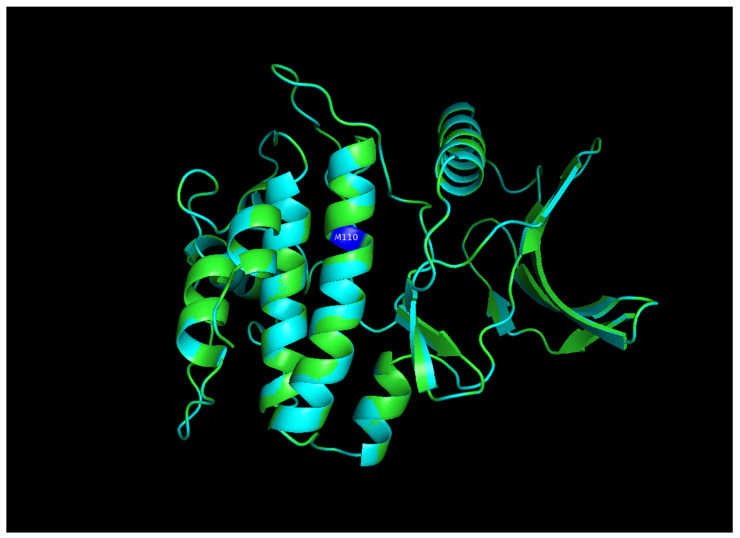
Structural Alignment pose of the predicted structure of normal and mutated NUAK2 proteins. The blue color is the mutated site, Methionine overlapped with Valine in the normal protein. RMSD obtained was 0.012 Å (1736 atoms).

On another ATP binding site at position 28, consisting of a Lysine residue, ATP binding was observed at lower energies in the mutated protein (−9.452 kcal/mol) than in the normal protein (−8.088 kcal/mol). However, as per the reported studies, methionine alterations in kinases tend to enhance their enzymatic activity and impart the protein more flexibility due to its unbranched side chain (Figure 3). On performing the protein structure analysis of both proteins using webPSN, it was also found that the hub proteins, i.e., the total number of nodes with at least four links, are 35 in the normal protein and 37 in the mutated NUAK2 protein.

## 3. Discussion

NTDs are considered complex diseases due to the interactions between genetic and environmental factors. Considering the multifactorial etiopathogenesis of this group of conditions, genes involved in folate and homocysteine metabolism, including 5, 10-methylenetetrahydrofolate reductase, methionine synthase, methionine synthase reductase, and methylenetetrahydrofolate dehydrogenase-1, have been extensively studied.

Notably, biallelic maternal polymorphisms in these genes have shown a statistically significant association with an increased risk of conceiving fetuses with NTDs, prompting some gynecologists to recommend higher folic acid dosages in these women.

The current approach to investigating the cause of NTDs involves gathering a detailed medical history and genetic assessments, including standard karyotype and chromosomal microarray analyses after sampling (chorionic villus or amniotic fluid in the prenatal setting, peripheral blood after birth). Further molecular testing is rarely requested and, due to the prevalence of variants of uncertain clinical significance, these additional analyses should be carefully evaluated and discussed [26]. Exome sequencing is a useful tool, especially for non-specific phenotypes, and it allows the identification of causative variants in selected cases [27].

Although pathogenic variants in more than 200 genes associated with NTDs have been reported in animal models [28], only a limited number of genotype-phenotype correlations have been identified in humans (Table 2). According to the literature, monoallelic pathogenic variants in *VANGL1* (MIM*610132, VANGL PLANAR CELL POLARITY PROTEIN 1), *VANGL2* (MIM*600533, VANGL PLANAR CELL POLARITY PROTEIN 2), *TBXT* (MIM*601397, T-BOX TRANSCRIPTION FACTOR T), and *FUZ* (MIM*610622, FUZZY PLANAR CELL POLARITY PROTEIN) correlate with increased susceptibility for NTDs.

The *CCL2* (MIM + 158105, C-C Motif Chemokine Ligand 2) −2518A>G promoter polymorphism has been associated with an increased risk for spina bifida in pregnant women who were homozygous for this variant [29]. Chemokine receptors [29], including CCR2 (the CCL2 receptor), are expressed in neural progenitor cells in the brain and play a role in chemokine-directed migration during neurogenesis.

The association between biallelic causative variants in the *TRIM36* (MIM*609317, TRIPARTITE MOTIF-CONTAINING PROTEIN 36) and in the *NUAK2* (MIM*608131, NUAK FAMILY, SNF1-LIKE KINASE, 2) genes with anencephaly remains provisional, as it has been described in one family each [8,9].

In particular, Singh and colleagues [8] detected the homozygous missense variant c.1522C>A; p.(Pro508Thr) in the *TRIM36* gene in a fetus with anencephaly conceived by a couple of Indian cousins. The alteration, in a highly conserved position, is predicted to alter the domain’s conformation.

In 2020, Bonnard et al. reported anencephaly in three fetuses of two Turkish first-degree cousins [9]. In this case, trio-exome sequencing performed on the second fetus and parental DNA revealed the presence of the homozygous loss-of-function in-frame c.412_433delinsG variant in the *NUAK2* gene. Sanger sequencing further confirmed that the same variant was present in the third fetus of the same family, too [9].

**Table 2:** Prenatal and postnatal cases of NTDs with identified molecular causes and related segregations reported in the literature. Polymorphisms are not included. Biallelic variants reported in both affected and unaffected individuals of the same family were excluded. Cases carrying variants but without a reported phenotype were not included. The grey lines corresponding to individuals of the same family are shown in the same color. A.D.: autosomal dominant; A.R.: autosomal recessive; N.A.: not available.

Reference	Individual	Gene	Variant	Aminoacidic Change	Zigosity	Transmission	Geographic Origin	Consanguineity	Neural Tube Defect
Kibar, 2007 [30]	19yo female	*VANGL1*	c.821G>A	p.Arg274Gln	Heterozygous	A.D, susceptibility to	Italian	No	Myelomeningocele
Kibar, 2007 [30]	mother	*VANGL1*	c.821G>A	p.Arg274Gln	Heterozygous	A.D., susceptibility to	Italian	No	Vertebral Schisis
Kibar, 2007 [30]	maternal aunt	*VANGL1*	c.821G>A	p.Arg274Gln	Heterozygous	A.D., susceptibility to	Italian	No	Vertebral Schisis
Kibar, 2007 [30]	21yo female	*VANGL1*	c.983T>C	p.Met328Thr	Heterozygous	A.D., susceptibility to	N.A.	No	Myelomeningocele, Chiari II malformation, tethered spinal cord
Kibar, 2007 [30]	10yo female	*VANGL1*	c.715G>A	p.Val239Ile	Heterozygous	A.D., susceptibility to	Italian	No	Sacral agenesis with lipomyeloschisis, tethered spinal cord
Kibar, 2007 [30]	mother	*VANGL1*	c.715G>A	p.Val239Ile	Heterozygous	A.D., susceptibility to	Italian	No	None
Kibar, 2007 [30]	brother	*VANGL1*	c.715G>A	p.Val239Ile	Heterozygous	A.D., susceptibility to	Italian	No	Dorsal dermal sinus
Iliescu, 2014 [31]	male	*VANGL1*	c.542G>A	p.Arg181Gln	Heterozygous	A.D., susceptibility to	Italian	No	Myelomeningocele
Iliescu, 2014 [31]	mother	*VANGL1*	c.542G>A	p.Arg181Gln	Heterozygous	A.D., susceptibility to	Italian	No	None
Lei, 2010 [32]	Fetus 1	*VANGL2*	c.251C>T	p.Ser84Phe	Heterozygous	A.D., susceptibility to	Han Chinese	No	Holoprosencephaly
Lei, 2010 [32]	Fetus 2	*VANGL2*	c.1057C>T	p.Arg353Cys	Heterozygous	A.D., susceptibility to	Han Chinese	No	Anencephaly, spina bifida
Lei, 2010 [32]	Fetus 3	*VANGL2*	c.1310T>C	p.Phe437Ser	Heterozygous	A.D., susceptibility to	Han Chinese	No	Anencephaly
Kibar, 2011 [33]	Patient	*VANGL2*	c.403C>T	p.Arg135Trp	Heterozygous	A.D., susceptibility to	Italian	N.A.	Lumbo-sacral myelomenigocele, Chiari II malformation, and hydromyelia
Kibar, 2011 [33]	Mother	*VANGL2*	c.403C>T	p.Arg135Trp	Heterozygous	A.D., susceptibility to	Italian	N.A.	N.A.
Kibar, 2011 [33]	Patient	*VANGL2*	c.530G>A	p.Arg177His	Heterozygous	A.D., susceptibility to	Italian	N.A.	Diastematomyelia
Kibar, 2011 [33]	Patient	*VANGL2*	c.809G>A	p.Arg270His	Heterozygous	A.D., susceptibility to	Italian	N.A.	Hydrosyringomyelia, fibrolipoma of the filum terminalis
Kibar, 2011 [33]	Patient	*VANGL2*	c.724C>G	p.Leu242Val	Heterozygous	A.D., susceptibility to	Italian	N.A.	Lumbar myeolocystocele
Kibar, 2011 [33]	Mother	*VANGL2*	c.724C>G	p.Leu242Val	Heterozygous	A.D., susceptibility to	Italian	N.A.	N.A.
Kibar, 2011 [33]	Patient	*VANGL2*	c.724C>G	p.Leu242Val	Heterozygous	A.D., susceptibility to	Caucasian white American	N.A.	Myelomeningocele
Kibar, 2011 [33]	Patient	*VANGL2*	c.740C>T	p.Thr247Met	Heterozygous	A.D., susceptibility to	Caucasian white American	N.A.	Lipoma of the filum terminalis, tethered cord
Kibar, 2011 [33]	Patient	*VANGL2*	c.532G>A	p.Val178Ile	Heterozygous	A.D., susceptibility to	N.A.	N.A.	Lipoma and tethered cord
Kibar, 2011 [33]	Father	*VANGL2*	c.532G>A	p.Val178Ile	Heterozygous	A.D., susceptibility to	N.A.	N.A.	N.A.
Seo, 2011 [34]	Neonate	*FUZ*	c.115C>T	p.Pro39Ser	Heterozygous	A.D., susceptibility to	Italian	No	Myelomeningocele, Chiari II malformation
Seo, 2011 [34]	Female child	*FUZ*	c.1060G>T	p.Asp354Tyr	Heterozygous	A.D., susceptibility to	Italian	No	Myelomeningocele, Chiari II malformation
Seo, 2011 [34]	Father	*FUZ*	c.1060G>T	p.Asp354Tyr	Heterozygous	A.D., susceptibility to	Italian	No	None
Seo, 2011 [34]	Male	*FUZ*	c.1211G>A	p.Arg404Gln	Heterozygous	A.D., susceptibility to	Caucasian	No	Hemimyelomeningocele, diastematomyelia, Chiari II malformation
Seo, 2011 [34]	Father	*FUZ*	c.1211G>A	p.Arg404Gln	Heterozygous	A.D., susceptibility to	Caucasian	No	None
Mastromoro, 2025 [35]	Fetus 1	*CCL2*	MIM*601156:arr[GRCh37]17q12(32104747_ arr[GRCh37]17q12(32104747_32744698)x3pat	_	Heterozygous	A.D.	Italian	No	Anencephaly
Mastromoro, 2025 [35]	Fetus 2	*CCL2*	MIM*601156:arr[GRCh37]17q12(32104747_ arr[GRCh37]17q12(32104747_32744698)x3pat	_	Heterozygous	A.D.	Italian	No	Anencephaly
Mastromoro, 2025 [35]	Father	*CCL2*	MIM*601156:arr[GRCh37]17q12(32104747_ arr[GRCh37]17q12(32104747_32744698)x3pat	_	Heterozygous	A.D.	Italian	No	Dorsal dermal sinus
Postma, 2014 [36]	Stillborn	*TBXT*	c.796A>G	p.His171Arg	Homozygous	A.R.	N.A.	Yes	Sacral agenesis and abnormal ossification of all vertebral bodies
Postma, 2014 [36]	Neonate	*TBXT*	c.796A>G	p.His171A.R.g	Homozygous	A.R.	N.A.	Yes	Sacral and complete left renal agenesis, persistent cloaca with anal atresia, and vertical clefting of all vertebral bodies
Postma, 2014 [36]	Child	*TBXT*	c.796A>G	p.His171Ar.g	Homozygous	A.R.	N.A.	Yes	Sacral agenesis and abnormal ossification of all vertebral bodies
Postma, 2014 [36]	Child	*TBXT*	c.796A>G	p.His171Arg	Homozygous	A.R.	N.A.	Yes	Sacral agenesis and abnormal ossification of all vertebral bodies
Singh, 2017 [8]	Fetus	*TRIM36*	c.1522C>A	p.Pro508Thr	Homozygous	A.R.	Indian	Yes	Anencephaly
Bonnard, 2020 [9]	Fetus 1	*NUAK2*	c.412_433delinsG	_	Homozygous	A.R.	Turkish	Yes	Anencephaly
Bonnard, 2020 [9]	Fetus 2	*NUAK2*	c.412_433delinsG	_	Homozygous	A.R.	Turkish	Yes	Anencephaly
Bonnard, 2020 [9]	Fetus 3	*NUAK2*	c.412_433delinsG	_	Homozygous	A.R.	Turkish	Yes	Anencephaly
Bonnard, 2020 [9]	Mother	*NUAK2*	c.412_433delinsG	_	Heterozygous	A.R.	Turkish	Yes	None
Bonnard, 2020 [9]	Father	*NUAK2*	c.412_433delinsG	_	Heterozygous	A.R.	Turkish	Yes	None

*NUAK2*, a gene located on chromosome 1q32.1, encodes the SNF1/5’-adenosine monophosphate-activated protein kinase (AMPK)-related kinase. The NUAK2 protein resides in nuclear speckles and regulates tolerance to glucose starvation. Its expression is induced by CD95 or TNF-alpha and, by increasing the conversion of F-actin into G-actin, induces cell–cell detachment.

NUAK2 protein plays a key role in neural tube closure by phosphorylating the serine/threonine kinase LATS2, a mitotic regulator required for coordinating cell division [37], and by regulating the nuclear localization of YAP1, a critical downstream target in the Hippo signaling pathway [9].

Several studies documented the role of NUAK2 in regulating cell–cell and cell–matrix adhesion, apoptosis, cell proliferation, and differentiation [38,39,40,41,42]. Alterations in cell adhesion in the neuroepithelia, together with the loss of basal lamina components, are known to contribute to the development of neural tube defects [43,44,45]. Likewise, the dysregulation of cell proliferation and the premature differentiation of neural plate cells into neurons during the closure can lead to defects in this process [46].

In 2012, Ohmura et al. investigated the phenotype of *Nuak1* and *Nuak2* double mutant mice, revealing facial cleft (failure of optic fissure closure), spina bifida, and exencephaly in all the knock-out animals. While *Nuak1* mutants showed no NTDs, 40% of single *Nuak2* knock-out mice exhibited exencephaly [47]. The study specifically highlighted insufficient apical constriction and elongation of neuroepithelial cells in the rostral region during neurulation. In the caudal region, in double mutants, the paired dorsolateral hinge points failed to form due to the overexpression of *Shh*, which determines a ventralization of the neural plate, preventing the differentiation of dorsal cells [47].

The current results are in line with these preclinical observations: the case study presented shows five individuals from the same family with a sacral dimple, which is a sign of NTD-related disorders. Sacral dimple results from the failure of the superficial ectoderm to separate from the neural ectoderm, leading to a focal adhesion that extends from the skin surface to varying depths [48,49]. The identified sequence change in coding exon 3 of 7 of the *NUAK2* gene substitutes valine, a non-polar amino acid, with methionine, another non-polar amino acid, at codon 163 of the resulting protein. This amino acid substitution occurs in the protein kinase domain, spanning amino acids 53 to 303, potentially altering the catalytic domain of the NUAK2 protein. Methionine contains a hydrophobic side chain with sulfur (S), which can oxidize and potentially interfere with kinase-substrate binding, leading to altered activity. Unlike valine, methionine has an unbranched side chain, offering greater flexibility. In phosphatase enzymes, methionine substitution often disrupts enzyme activity, while in kinases, methionine substitution typically enhances activity.

The *NUAK2* c.487G>A variant, with a 0.000041% allele frequency in Exome Aggregation Consortium (ExAC), can be classified as “variant of unknown significance” according to the American College of Medical Genetics and Genomics guidelines for variant interpretation [24], and it is not reported in the ClinVar database [50]. We noted that the valine residue at codon 163 is highly conserved across vertebrates. The SIFT4G and LIST-S2 algorithms suggest that this missense variant is likely to be deleterious. The variant is not reported in ClinVar, and it can be classified as a variant of unknown significance according to the criteria of the American College of Medical Genetics and Genomics (PM2 moderate) [24]. GnomAD Frequency exomes: ƒ = 0.0000219. Genomes: ƒ = 0.0000263. The identified variant was predicted to be deleterious by several widely used in silico predictors, including Combined Annotation Dependent Depletion (CADD) v1.7 score 25.1 [25]; REVEL score 0.246; phyloP score 5.00; PolyPhen2 score 0.907; PaPI score 1.

The haploinsufficiency intolerance of the identified c.487G>A missense variant, as predicted by protein-ligand interaction (pLI), was 3.18 × 10^−5^, supporting its effect in heterozygosity. The ExAC score for the missense variant was 1.5, indicating increased constraint (intolerance to variation), suggesting that the gene harbors fewer variants than expected.

Notably, the current identified c.487G>A missense variant occurs in the same protein kinase domain (c.412_433delinsG) as the previously reported homozygous variant in *NUAK2* associated with anencephaly (MIM #619452; ANPH2) [9].

This is the first report of spinal dysraphism segregating in a family with a monoallelic variant in *NUAK2*. Biallelic pathogenic variants in this gene have been reported in association with anencephaly, the most severe phenotype in the spectrum of neural tube defects. The present report expands the knowledge of monogenic causes of neural tube defects and suggests that *NUAK2* variants may exhibit a semidominant behavior, with mild clinical features in individuals carrying a heterozygous variant and severe phenotypes when biallelic variants determine a complete loss of function of the resulting protein. This report lays the foundation for new lines of research that may allow easier identification of individuals with monoallelic variants in *NUAK2*, access to molecular investigations, and, in the case of a couple carrying variants in this gene, to discuss preimplantation genetic diagnosis with embryo selection.

Recognizing clinical phenotypes associated with heterozygous variants in the *NUAK2* gene can substantially modify reproductive risks, allowing for more appropriate pregnancy planning and management.

## 4. Conclusions

The present report corroborates the association between loss-of-function variants in *NUAK2* and NTDs. Identifying individuals with an a priori increased risk of conceiving fetuses with a Mendelian form of anencephaly is crucial for effective genetic counseling.

## Figures and Tables

**Table 1:** Tabulating the results of docking analysis performed using the GLIDE module of Maestro Schrodinger software 13.1 version. Receptor proteins involved are normal and mutated NUAK2 proteins, and the ligand used is ATP. * Solid pink lines represent H bonds to the protein backbone.

N.	Site of Docking	Docking Residues Position	Docking Score (kcal/mol)	3D Structure	2D Structure *	Interacting Residues	Interaction Type
1.	6–14 (LGKGTYGKV) Ligand ATP GRID coordinates: 0.542(X) −3.736(y) 17.467(Z)	NUAK2	−10.595	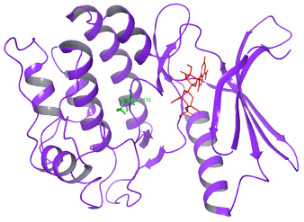	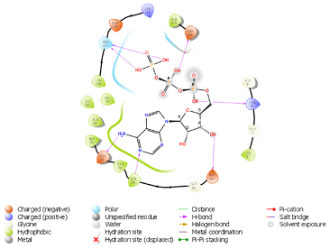	Lys:8, Asp:83, Ala:79, Glu:77, Asn:127, Asp:140	H-bond to protein backbone
		Mutated NUAK2	−8.881	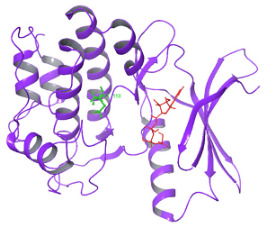	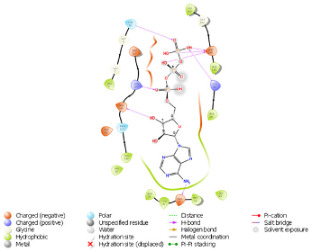	Thr:10, Lys:28, Glu:77, Lys:124, Glu:126, Asp:140	H-bond to protein backbone
2.	28 (K) Ligand ATP GRID coordinates: −4.107(X) −0.247(y) 14.504(z)	NUAK2	−8.088	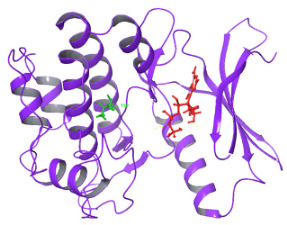	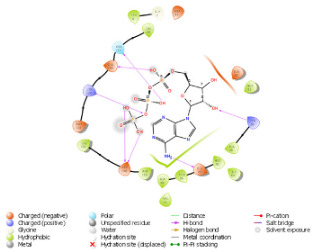	Lys:28, Glu:77, Lys:124, Glu:126, Asn:127	H-bond to protein backbone
		Mutated NUAK2	−9.452	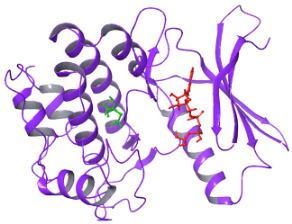	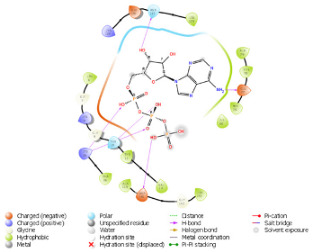	Thr:10, Lys:28, Glu:77, Asn:127, Asp:140	H-bond to protein backbone

## Data Availability

Data is available from the corresponding authors after reasonable requests.

## References

[1] Van Allen M.I., Kalousek D.K., Chernoff G.F., Juriloff D., Harris M., McGillivray B.C., Yong S.L., Langlois S., MacLeod P.M., Chitayat D. (1993). Evidence for multi-site closure of the neural tube in humans. Am. J. Med. Genet..

[2] Rossi A.C., Prefumo F. (2013). Accuracy of ultrasonography at 11–14 weeks of gestation for detection of fetal structural anomalies. Obstet. Gynecol..

[3] Mastromoro G., Guadagnolo D., Khaleghi Hashemian N., Bernardini L., Giancotti A., Piacentini G., De Luca A., Pizzuti A. (2022). A Pain in the Neck: Lessons Learnt from Genetic Testing in Fetuses Detected with Nuchal Fluid Collections, Increased Nuchal Translucency versus Cystic Hygroma-Systematic Review of the Literature, Meta-Analysis and Case Series. Diagnostics.

[4] Thi Pham X.T., Nguyen P.N., Hoang X.S. (2025). Fraser Syndrome: A Narrative Review Based on a Case from Vietnam and the Past 20 Years of Research. Diagnostics.

[5] Buijtendijk M.F., Bet B.B., Leeflang M.M., Shah H., Reuvekamp T., Goring T., Docter D., Timmerman M.G., Dawood Y., Lugthart M.A. (2024). Diagnostic accuracy of ultrasound screening for fetal structural abnormalities during the first and second trimester of pregnancy in low-risk and unselected populations. Cochrane Database Syst. Rev..

[6] Dulgheroff F.F., Peixoto A.B., Petrini C.G., Caldas T.M.R.D.C., Ramos D.R., Magalhães F.O., Araujo Júnior E. (2019). Fetal structural anomalies diagnosed during the first, second and third trimesters of pregnancy using ultrasonography: A retrospective cohort study. Sao Paulo Med. J..

[7] Detrait E.R., George T.M., Etchevers H.C., Gilbert J.R., Vekemans M., Speer M.C. (2005). Human neural tube defects: Developmental biology, epidemiology, and genetics. Neurotoxicol. Teratol..

[8] Singh N., Kumble Bhat V., Tiwari A., Kodaganur S.G., Tontanahal S.J., Sarda A., Malini K.V., Kumar A. (2017). A homozygous mutation in TRIM36 causes autosomal recessive anencephaly in an Indian family. Hum. Mol. Genet..

[9] Bonnard C., Navaratnam N., Ghosh K., Chan P.W., Tan T.T., Pomp O., Ng A.Y.J., Tohari S., Changede R., Carling D. (2020). A loss-of-function NUAK2 mutation in humans causes anencephaly due to impaired Hippo-YAP signaling. J. Exp. Med..

[10] Copp A.J., Stanier P., Greene N.D. (2013). Neural tube defects: Recent advances, unsolved questions, and controversies. Lancet Neurol..

[11] Moldenhauer J.S., Adzick N.S. (2017). Fetal surgery for myelomeningocele: After the Management of Myelomeningocele Study (MOMS). Semin. Fetal Neonatal Med..

[12] Adzick N.S., Thom E.A., Spong C.Y., Brock J.W., Burrows P.K., Johnson M.P., Howell L.J., Farrell J.A., Dabrowiak M.E., Sutton L.N. (2011). A randomized trial of prenatal versus postnatal repair of myelomeningocele. N. Engl. J. Med..

[13] Belfort M.A., Whitehead W.E., Shamshirsaz A.A., Ruano R., Cass D.L., Olutoye O.O., Mann A.G., Espinoza J., Williams E., Lee T.C. (2017). Fetoscopic open neural tube defect repair: Development and refinement of a two-port, carbon dioxide insufflation technique. Ultrasound Obstet. Gynecol..

[14] Hall J.G., Friedman J.M., Kenna B.A., Popkin J., Jawanda M., Arnold W. (1988). Clinical, genetic, and epidemiological factors in neural tube defects. Am. J. Hum. Genet..

[15] Rampersaud E., Melvin E.C., Speer M.C., Wyszynski D.F. (2006). Nonsyndromic neural tube defects: Genetic basis and genetic investigations. Neural Tube Defects: From Origin to Treatment.

[16] Seller M.J. (1994). Risks in spina bifida. Dev. Med. Child Neurol..

[17] Hibbard B.M., Hibbard E.D., Jeffcoate T.N. (1965). Folic acid and reproduction. Acta Obs. Gynecol. Scand..

[18] MRC Vitamin Study Research Group (1991). Prevention of neural tube defects: Results of the Medical Research Council Vitamin Study. Lancet.

[19] Greene N.D., Leung K.Y., Copp A.J. (2017). Inositol, neural tube closure and the prevention of neural tube defects. Birth Defects Res..

[20] Greene N.D., Leung K.Y., Gay V., Burren K., Mills K., Chitty L.S., Copp A.J. (2016). Inositol for the prevention of neural tube defects: A pilot randomised controlled trial. Br. J. Nutr..

[21] Carter C.O., Evans K.A. (1973). Spina bifida and anencephalus in Greater London. J. Med. Genet..

[22] Elwood J.H., Nevin N.C. (1973). Factors associated with anencephalus and spina bifida in Belfast. Br. J. Prev. Soc. Med..

[23] Berry R.J., Li Z., Erickson J.D., Li S., Moore C.A., Wang H., Mulinare J., Zhao P., Wong L.Y., Gindler J. (1999). Prevention of neural-tube defects with folic acid in China. China-U.S. Collaborative Project for Neural Tube Defect Prevention. N. Engl. J. Med..

[24] Richards S., Aziz N., Bale S., Bick D., Das S., Gastier-Foster J., Grody W.W., Hegde M., Lyon E., Spector E. (2015). Standards and guidelines for the interpretation of sequence variants: A joint consensus recommendation of the American College of Medical Genetics and Genomics and the Association for Molecular Pathology. Genet. Med. Off. J. Am. Coll. Med. Genet..

[25] Schubach M., Maass T., Nazaretyan L., Röner S., Kircher M. (2024). CADD v1.7: Using protein language models, regulatory CNNs and other nucleotide-level scores to improve genome-wide variant predictions. Nucleic Acids Res..

[26] Guadagnolo D., Mastromoro G., Di Palma F., Pizzuti A., Marchionni E. (2021). Prenatal Exome Sequencing: Background, Current Practice and Future Perspectives-A Systematic Review. Diagnostics.

[27] Mastromoro G., Guadagnolo D., Khaleghi Hashemian N., Marchionni E., Traversa A., Pizzuti A. (2022). Molecular Approaches in Fetal Malformations, Dynamic Anomalies and Soft Markers: Diagnostic Rates and Challenges-Systematic Review of the Literature and Meta-Analysis. Diagnostics.

[28] Harris M.J., Juriloff D.M. (2010). An update to the list of mouse mutants with neural tube closure defects and advances toward a complete genetic perspective of neural tube closure. Birth Defects Res. Part A Clin. Mol. Teratol..

[29] Jensen L.E., Etheredge A.J., Brown K.S., Mitchell L.E., Whitehead A.S. (2006). Maternal genotype for the monocyte chemoattractant protein 1 A(-2518)G promoter polymorphism is associated with the risk of spina bifida in offspring. Am. J. Med. Genet. A.

[30] Kibar Z., Torban E., McDearmid J.R., Reynolds A., Berghout J., Mathieu M., Kirillova I., De Marco P., Merello E., Hayes J.M. (2007). Mutations in VANGL1 associated with neural-tube defects. N. Engl. J. Med..

[31] Iliescu A., Gravel M., Horth C., Gros P. (2014). Independent mutations at Arg181 and Arg274 of Vangl proteins that are associated with neural tube defects in humans decrease protein stability and impair membrane targeting. Biochemistry.

[32] Lei Y.P., Zhang T., Li H., Wu B.L., Jin L., Wang H.Y. (2010). VANGL2 mutations in human cranial neural-tube defects. N. Engl. J. Med..

[33] Kibar Z., Salem S., Bosoi C.M., Pauwels E., De Marco P., Merello E., Bassuk A.G., Capra V., Gros P. (2011). Contribution of VANGL2 mutations to isolated neural tube defects. Clin. Genet..

[34] Seo J.H., Zilber Y., Babayeva S., Liu J., Kyriakopoulos P., De Marco P., Merello E., Capra V., Gros P., Torban E. (2011). Mutations in the planar cell polarity gene, Fuzzy, are associated with neural tube defects in humans. Hum. Mol. Genet..

[35] Mastromoro G., Darelli D., Mariani S., Colantoni F., Bucossi S., Canestrelli M., Russo C.D., Squitti R., Micalizzi A., Zumpano A. (2025). Microduplication encompassing CCL2 segregates in a family with recurrent anencephaly and dorsal dermal sinus: A possible link between chemokine and neural tube defects?. Eur. J. Obstet. Gynecol. Reprod. Biol..

[36] Postma A.V., Alders M., Sylva M., Bilardo C.M., Pajkrt E., van Rijn R.R., Schulte-Merker S., Bulk S., Stefanovic S., Ilgun A. (2014). Mutations in the T (brachyury) gene cause a novel syndrome consisting of sacral agenesis, abnormal ossification of the vertebral bodies and a persistent notochordal canal. J. Med. Genet..

[37] Yabuta N., Okada N., Ito A., Hosomi T., Nishihara S., Sasayama Y., Fujimori A., Okuzaki D., Zhao H., Ikawa M. (2007). Lats2 is an essential mitotic regulator required for the coordination of cell division. J. Biol. Chem..

[38] Suzuki A., Kusakai G., Kishimoto A., Minegichi Y., Ogura T., Esumi H. (2003). Induction of cell-cell detachment during glucose starvation through F-actin conversion by SNARK, the fourth member of the AMP-activated protein kinase catalytic subunit family. Biochem. Biophys. Res. Commun..

[39] Legembre P., Schickel R., Barnhart B.C., Peter M.E. (2004). Identification of SNF1/ AMP kinase-related kinase as an NFkappaB-regulated anti-apoptotic kinase involved in CD95-induced motility and invasiveness. J. Biol. Chem..

[40] Suzuki A., Kusakai G., Kishimoto A., Shimojo Y., Miyamoto S., Ogura T., Ochiai A., Esumi H. (2004). Regulation of caspase-6 and FLIP by the AMPK family member ARK5. Oncogene.

[41] Hou X., Liu J.E., Liu W., Liu C.Y., Liu Z.Y., Sun Z.Y. (2011). A new role of NUAK1: Directly phosphorylating p53 and regulating cell proliferation. Oncogene.

[42] Namiki T., Tanemura A., Valencia J.C., Coelho S.G., Passeron T., Kawaguchi M., Vieira W.D., Ishikawa M., Nishijima W., Izumo T. (2011). AMP kinase-related kinase NUAK2 affects tumor growth, migration, and clinical outcome of human melanoma. Proc. Natl. Acad. Sci. USA.

[43] Miner J.H., Cunningham J., Sanes J.R. (1998). Roles for laminin in embryogenesis: Exencephaly, syndactyly, and placentopathy in mice lacking the laminin alpha5 chain. J. Cell Biol..

[44] Arikawa-Hirasawa E., Watanabe H., Takami H., Hassell J.R., Yamada Y. (1999). Perlecan is essential for cartilage and cephalic development. Nat. Genet..

[45] Morita H., Nandadasa S., Yamamoto T.S., Terasaka-Iioka C., Wylie C., Ueno N. (2010). Nectin-2 and N-cadherin interact through extracellular domains and induce apical accumulation of F-actin in apical constriction of Xenopus neural tube morphogenesis. Development.

[46] Copp A.J., Greene N.D., Murdoch J.N. (2003). The genetic basis of mammalian neurulation. Nat. Rev. Genet..

[47] Ohmura T., Shioi G., Hirano M., Aizawa S. (2012). Neural tube defects by NUAK1 and NUAK2 double mutation. Dev. Dyn..

[48] Radswiki T., Walizai T., Gaillard F. Dorsal Dermal Sinus. https://radiopaedia.org/articles/dorsal-dermal-sinus?lang=us.

[49] Naidich T.P., Zimmerman R.A., McLone D.G., Raybaud C.A., Altman N.R., Braffman B.H., Atlas S.W. (1996). Congenital anomalies of the spine and spinal cord. Magnetic Resonance Imaging of the Brain and Spine.

[50] Landrum M.J., Lee J.M., Benson M., Brown G.R., Chao C., Chitipiralla S., Gu B., Hart J., Hoffman D., Jang W. (2018). ClinVar: Improving access to variant interpretations and supporting evidence. Nucleic Acids Res..

